# 

**Published:** 1885-04

**Authors:** 


### Illinois State Dental Society.

The twenty-first annual meeting of the Illinois State Dental Society will be held at Peoria, Ill., commencing Tuesday, May 12th, 1885, and continuing four days.

The State Board of Dental Examiners will be at the National Hotel at 10 A. M., Monday, May 11th, at which time candidates for examination must present themselves punctually.

The examinations will continue until Thursday, May 14th.

J. W. WASSALL, Sec'y.
103 State st., Chicago.

---

THE twenty-third annual meeting of the *Iowa State Dental Society* will be held in Des Moines beginning on the first Tuesday in May continuing four days.

Officers.—President, S. A. Garber, Tipton; Vice-President, L. E. Rogers, Ottumwa; Secretary, J. B. Monfort, Fairfield; Treasurer, J. S. Kulp, Muscatine.

Executive Committee.—G. W. Fultes, Des Moines; H. A. Woodbury, Council Bluffs; E. E. Hughes, Des Moines.

Every effort is being made to present an interesting and instructive programme. A large attendance is expected. A cordial welcome will be given to members of the profession from other states.

J. B. MONFORT, Secretary.

---

### Mad River Valley Dental Society.

Meets in annual session for *one day only*, in the parlors of the Phillips House, Dayton, Tuesday, May 19, 1885.

Sessions begin at 10 A. M., 2 and 7 P. M.

No routine business. Whole time devoted to papers and discussions of topics.

### CONTENTS.

### THE DENTAL REGISTER,

A MONTHLY MAGAZINE DEVOTED TO THE INTERESTS OF THE

**DENTAL PROFESSION.**

*Terms Reduced to* $2.00 *Per Year. Single Copies,* 25 *Cents.*

**EDITED BY PROF. J. TAFT, D.D.S.**

—AND—

**Published by SPENCER & CROCKER, Ohio Dental and Surgical Depot.**

The volume commences in January and closes in December.

The REGISTER being issued monthly is always fresh, therefore Indispensable to the practitioner who desires to keep posted up with the new inventions and improvements which are daily developed. Neither expense nor effort will be spared to give value and variety to its contents. Articles will be illustrated with well executed engravings whenever they will add to the interest or assist to explanation.

Its extensive circulation and frequency of issue make it one of the BEST DENTAL ADVERTISING MEDIUMS in the United States.

All subscriptions must begin with January or July.

Local or special notices, five lines or less, will be inserted for $3 each insertion; over five lines, 50 cts. per line.

All advertising bills payable in advance, or at furthest, after the issue of the first number containing the advertisement. All transient advertisements to be paid for in advance.

☞CONTRIBUTIONS SOLICITED.

Exclusively Editorial Communications should be addressed to the Editor.

The latest number of the REGISTER may be ordered with or without other goods at any time, and all letters should be addressed to

**SPENCER & CROCKER, Ohio Dental & Surgical Depot, Cincinnati, O.**

